# Testicular volume in adolescent varicocele

**DOI:** 10.4103/0971-3026.76064

**Published:** 2011

**Authors:** Ramen Kumar Baishya, Divya Ratna Dhawan, Ravindra Sabnis, Mahesh R Desai

**Affiliations:** Department of Urology, Muljibhai Patel Urological Hospital, Nadiad, Gujarat, India E-mail: mrdesai@mpuh.org

Dear Sir,

Adolescent varicocele is a common finding. 7.8-14.1% of male adolescents are found to have varicoceles, which are mostly left sided and asymptomatic.[1] Prophylactic management of varicocele in adolescents lacks literature support, although a decline in the testicular volume over time of >20% calls for intervention.[2] The routine use of an orchidometer to determine testicular growth impairment is limited by its relative insensitivity to assess precise volume variations. USG is a viable alternative and preferred modality for a routine annual follow-up, as it can accurately record sequential growth alterations.[3] We retrospectively evaluated the last 100 patients of adolescent varicocele referred to us. We found that the testicular volume was not available in the USG reports of 71% of these patients. Repeating the USG increases costs.

I have written this letter with the hope of raising awareness among radiologists and ultrasonologists of the need to record testicular volumes in all adolescents who present with varicocele in their USG reports.
